# Protective effects of low-molecular-weight components of adipose stem cell-derived conditioned medium on dry eye syndrome in mice

**DOI:** 10.1038/s41598-021-01503-z

**Published:** 2021-11-08

**Authors:** Yuan-Chieh Lee, Li-Yi Sun, Jia-Rong Zhang

**Affiliations:** 1 Department of Ophthalmology, Hualien Tzu Chi Hospital, Buddhist Tzu Chi Medical Foundation, 707 Sec. 3 Chung-Yung Road, Hualien, 97002 Taiwan; 2 Department of Ophthalmology and Visual Science, School of Medicine, Tzu Chi University, Hualien, Taiwan; 3 Institute of Medical Sciences, Tzu Chi University, Hualien, Taiwan; 4 Department of Research, Hualien Tzu Chi Hospital, Buddhist Tzu Chi Medical Foundation, Hualien, Taiwan

**Keywords:** Medical research, Eye diseases

## Abstract

The present study demonstrated the protective effects of low-molecular-weight adipose-derived stem cell-conditioned medium (LADSC-CM) in a mouse model of dry eye syndrome. Mice subjected to desiccating stress and benzalkonium chloride had decreased tear secretion, impaired corneal epithelial tight junction with microvilli, and decreased conjunctival goblet cells. Topical application of adipose-derived stem cell-conditioned medium (ADSC-CM) stimulated lacrimal tear secretion, preserved tight junction and microvilli of the corneal epithelium, and increased the density of goblet cells and MUC16 expression in the conjunctiva. The low-molecular-weight fractions (< 10 kDa and < 3 kDa) of ADSC-CM (LADSC-CM) provided better protections than the > 10 kDa or > 3 kDa fractions of ADSC-CM. In the in vitro study, desiccation for 10 min or hyperosmolarity (490 osmols) for 24 h caused decreased viability of human corneal epithelial cells, which were reversed by LADSC-CM. The active ingredients in the LADSC-CM were lipophobic and stable after heating and lyophilization. Our study demonstrated that LADSC-CM had beneficial effects on experimental dry eye. It is worthy of further exploration for the active ingredient(s) and the mechanism.

## Introduction

Dry eye is a failure of homeostasis of the tear film due to inadequate production, malfunction, or excessive loss of tear components, including mucin, aqueous, and lipid^1^. The central mechanism is lack of hydration and hyperosmolar tissue damage^2^, but also involves inflammation and neurosensory abnormalities^3,4^. Reduced tear secretion leads to inflammation and peripheral nerve damage^4^, while neural degeneration or injury leads to further decreased tear production, forming a vicious cycle. The prevalence of dry eye is increasing worldwide^5–7^. Common associated factors include age^5–14^, female gender^7,12–15^, extended visual display terminal use^8,16–20^, sleep disorder^21,22^, environmental factors^9,12,17,23,24^, seasonality^24^, etc., among which age is the most important and universal^10^.

Current treatments for dry eyes include lubricants or tear supplements for lacking components such as sodium hyaluronate and diquafosol, anti-inflammatory drugs such as corticosteroids or cyclosporine, epithelial growth factor, autologous serum, platelet lysate (or platelet-rich plasma), or/and punctal occlusion, etc^25^. Among these treatments, only diquafosol, a P2Y2 agonist, claims to increase aqueous, lipid, and mucin components of tear production^25^. Autologous serum provides lubrication and some biochemical features mimicking natural tears but has only limited conclusions about its effects on symptoms and signs of dry eye^26^. Punctal occlusion decreases tear outflow but increases the concentration of inflammatory mediators in the tear film^27^, hence its role in dry eye treatment is inconclusive^28^. Holland et al. reviewed twenty-six trials investigating thirteen ophthalmic drugs for dry eye and described “None of the large (N > 100) studies demonstrated statistical significance of primary endpoints for both a sign and a symptom endpoint versus a control treatment in the same published trial”^29^. Therefore, further investigation for better treatment is warranted for this unmet need.

Stem cells are thought promising in degenerative disorders, and their roles in the dry eye have been investigated. Intravenous injection of bone marrow-derived mesenchymal stem cells (BMSC) was reported beneficial for the clinical symptoms in patients with refractory dry eye secondary to GVHD^30^. Topical application of BMSC showed some advantages in a rat benzalkonium chloride-induced dry eye syndrome^31^. Periorbital administration of BMSC induced aqueous tear production and increased the number of conjunctival goblet cells in a mice concanavalin A-induced inflammatory dry eye model^32^. Topical application of adipose-derived mesenchymal stem cells was reported to reduce the inflammatory markers CD4, IL-1, IL-6, and TNFα in dogs with keratoconjunctivitis sicca^33^.

Apart from the stem cells, the paracrine factors released from stem cells also enhance tissue regeneration and alleviate inflammation^34–37^. Human uterine cervical stem cells-conditioned medium has been reported to help rat corneal epithelial cells regeneration^38,39^. In contrast, adipose-derived stem cells (ADSCs)-conditioned medium (ADSC-CM) was found to contain growth factors such as VEGF, FGF-2, HGF, G-CSF, GM-CSF, IL-6, KGF, VEGF, TGF-β3, SDF-1a, etc^40,41^. ADSC-CM has been reported to speed recovery from liver diseases^42,43^, protect photoaging of the skin^44^, promote hair growth^45^, etc^46^. Human ADSC derived extracellular vesicles (size about 100 nm) eye drops have recently been shown to alleviate ocular surface damage in a mouse model of dry eye disease^47^. In this study, we demonstrated the protective effects of ADSC-CM on a mouse model of dry eye, which were attributed to the low-molecular-weight components (< 3 kD) in ADSC-CM (LADSC-CM) containing small-sized molecules (< 2 nm) that are far smaller than extracellular vesicles such as exosomes (with a size range 30–150 nm).

## Results

**Figure 1:**
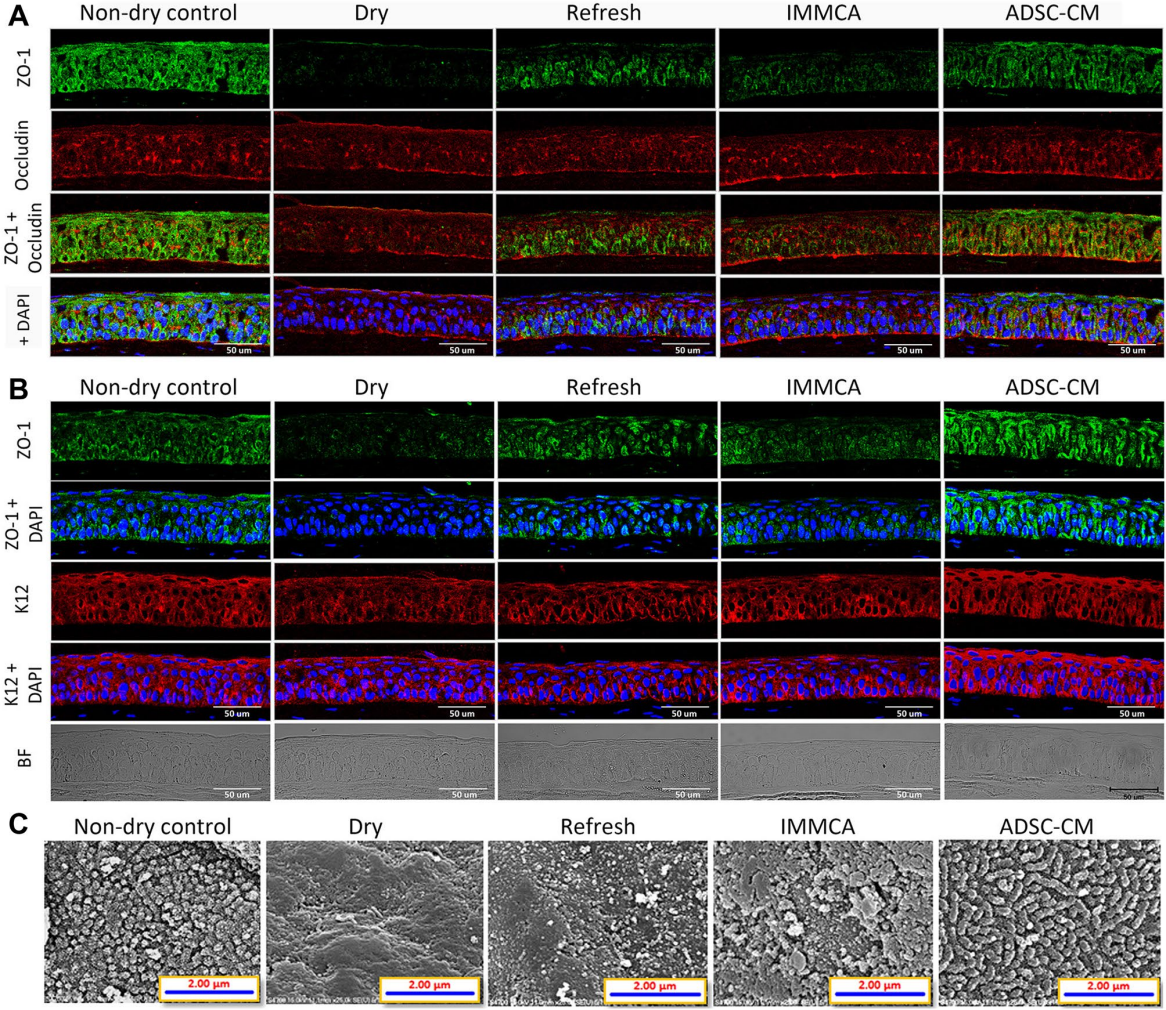
Confocal microscopic examination of the tight junction and scanning electron microscopy of corneal epithelium of BALB/c mice in the controlled-environment chamber (CEC)-induced dry eye model. **(A)** ZO-1 and occludin expression was suppressed in the BALB/c mice from CEC. Although topical application of Refresh Plus lubricant eye drops or IMMCA alleviated the suppressed expression caused by dry stress, ADSC-CM showed the best rescue. **(B)** In another experiment, ZO-1 and K12 expressions were also suppressed by dry stress, and the best expression was demonstrated in the ADSC-CM group. **(C)** Scanning electron microscopy of cornea demonstrated that the microvilli of corneal epithelium were lost in the dry eye mice. Topical application of Refresh Plus lubricant eye drops or IMMCA partially preserved the microvilli of corneal epithelium, while ADSC-CM protected the microvilli best from dry damage. Magnification: ×25,000.

**Figure 2:**
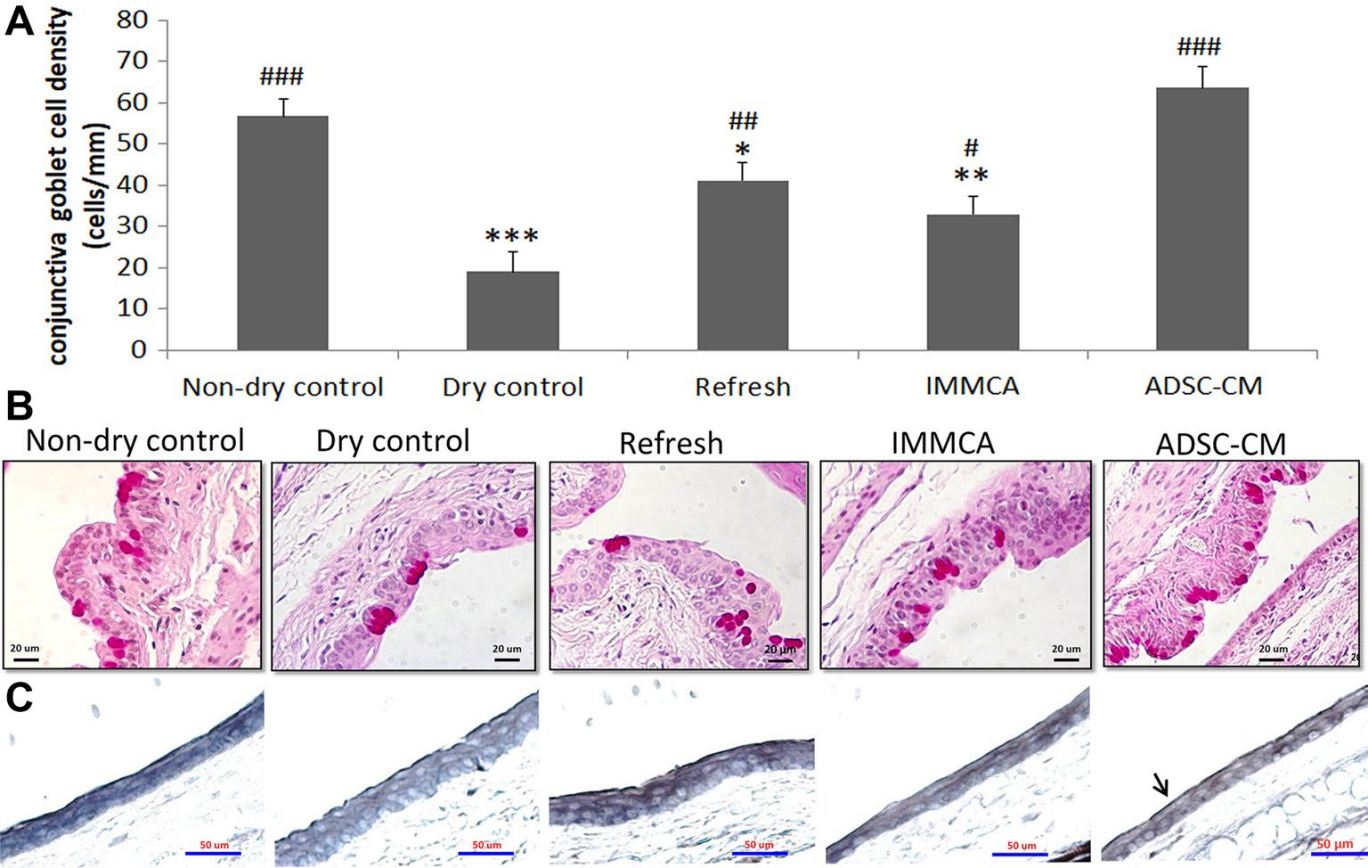
Conjunctival goblet cell density and MUC16 expression of the BALB/c mice in the controlled-environment chamber (CEC)-induced dry eye model. **(A,B)** Periodic acid–Schiff staining showed that conjunctival goblet cells were reduced in the CEC-induced dry eye. Refresh Plus lubricant eye drops and Iscove’s modified Dulbecco’s medium supplemented with mesenchymal stem cells culture adjuvant (IMMCA) partially reversed the reduction, while ADSC-CM best preserved the density of goblet cells. Data were presented as means ± SD. *p < 0.05, **p < 0.01, ***p < 0.001 compared with non-dry control. #p < 0.05, ##p < 0.01, ###p < 0.001 compared with dry control group. (N = 5). **(C)** Immunohistochemical analysis of MUC16 showed that conjunctival MUC16 expression was continuous in the non-dry group (99% continuity) but disrupted in the dry group (89% continuity), Refresh Plus group (90% continuity), and IMMCA group (90% continuity). Topical application of ADSC-CM in the CEC-dry eye mice helped keep the continuous expression of MUC16 (97% continuity).

In a controlled-environment chamber (CEC)^48–50^ combined with benzalkonium chloride (BAC)^51–53^ BALB/c mice dry eye model, topical application of ADSC-CM showed better protection than Refresh Plus lubricant eye drops (Allergan, Westport, Ireland), Iscove’s modified Dulbecco’s medium supplemented with glutamine, 10% fetal bovine serum, and mesenchymal stem cells culture adjuvant (abbreviated as IMMCA, which was the same medium as ADSC-CM but not conditioned by ADSCs). The confocal microscopy study of corneal epithelium revealed a decreased expression of zonula occludens-1 (ZO-1), occludin, and keratin 12 (K12) in the CEC-induced dry eye. The impaired expression was partially reversed by Refresh Plus and IMMCA, but the best expression was noted in the ADSC-CM group (Fig. 1A,B). The scanning electron microscopy (SEM) study demonstrated that the microvilli of corneal epithelium were lost in the CEC-induced dry eye mice. There were some preserved microvilli but also with bare area in the group treated with Refresh Plus and IMMCA, while most microvilli were well maintained in the group treated with ADSC-CM (Fig. 1C). Periodic acid-Schiff (PAS) staining showed that conjunctival goblet cells were reduced in the CEC-induced dry eye. Refresh Plus and IMMCA partially reversed the reduction, while ADSC-CM best preserved the density of goblet cells (Fig. 2A,B). The immunohistochemical study of MUC16 also showed that the ADSC-CM group had the best expression of MUC16 (Fig. 2C).

**Figure 3:**
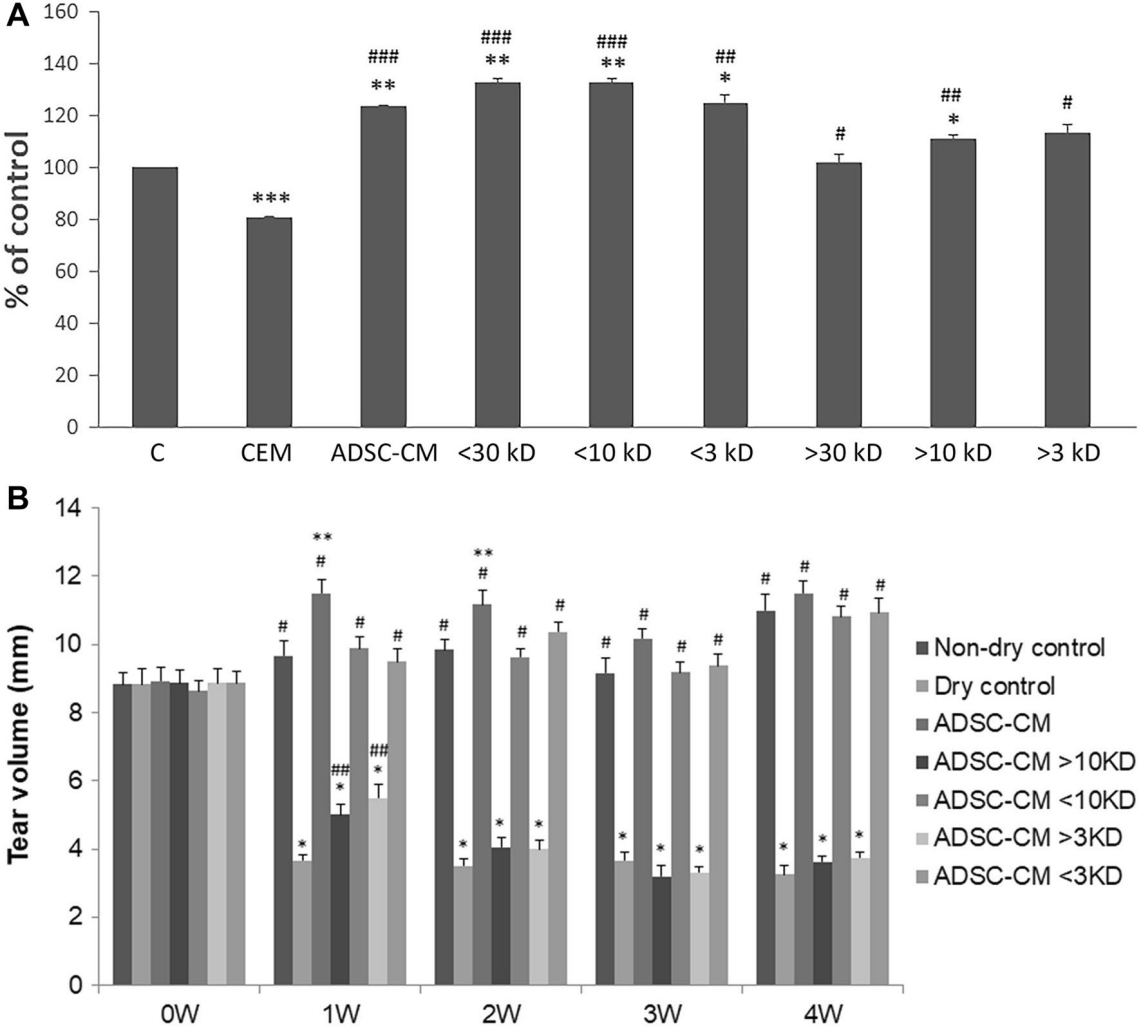
Different size fractions of ADSC-CM showed different protective effects in the HCEC desiccation stress experiment and tear stimulation effects in the controlled-environment chamber (CEC) mice. **(A)** HCECs treated with fractions of molecular size < 30 kD, < 10 kD, < 3kD after desiccation showed better viability than thosed treated with > 30 kD, > 10 kD, and > 3 kD. (N = 3) *p < 0.05, **p < 0.01, ***p < 0.001 compared with the non-dry control. #p < 0.05, ##p < 0.01, ### p < 0.001 compared with the corneal epithelial cell basal medium (CEM). (N = 5) **(B)** In the CEC-induced dry eye study, mice reated with fractions ADSC-CM with molecular size < 10 kD or < 3kD had more tear secretion than thosed treated with > 10 kD or > 3 kD. (N = 4). *p < 0.05, **p < 0.01, *** *p* < 0.001, compared with Non-dry control. ^#^*p* < 0.05, ^# #^ *p* < 0.01, ^# # #^ *p* < 0.001, compared with Dry control. The values were expressed as the means ± SD. (N = 5).

**Figure 4:**
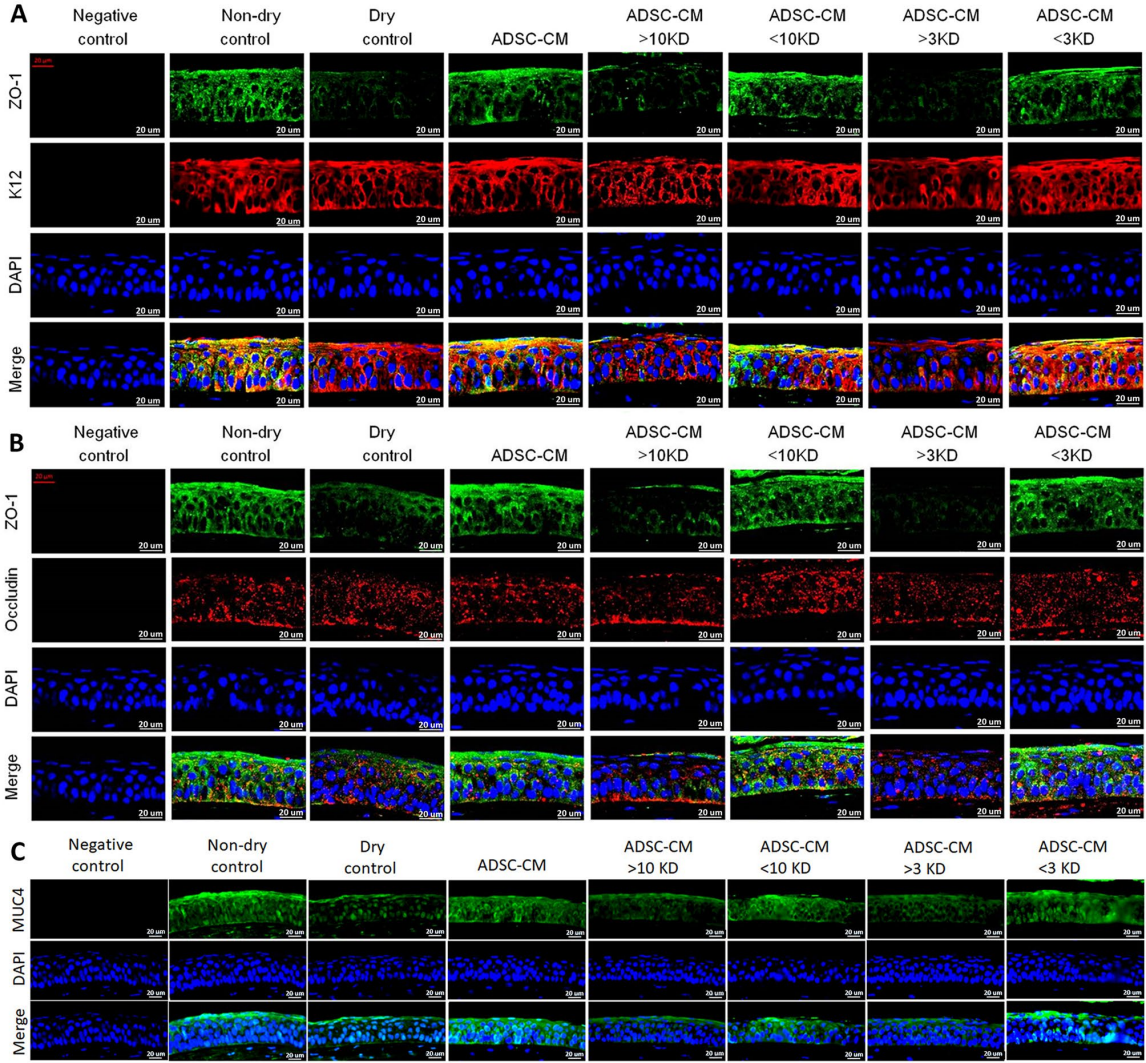
Confocal examination of tight junction and MUC4 expression of the corneal epithelium in the CEC mice. Mice treated with fractions of ADSC-CM with molecular size < 10 kD or < 3kD showed better expression of **(A)** ZO-1 and K12, **(B)** ZO-1 and occludin, and **(C)** MUC4 than those treated with fractions > 10 kD or > 3 kD. (N = 3).

**Figure 5:**
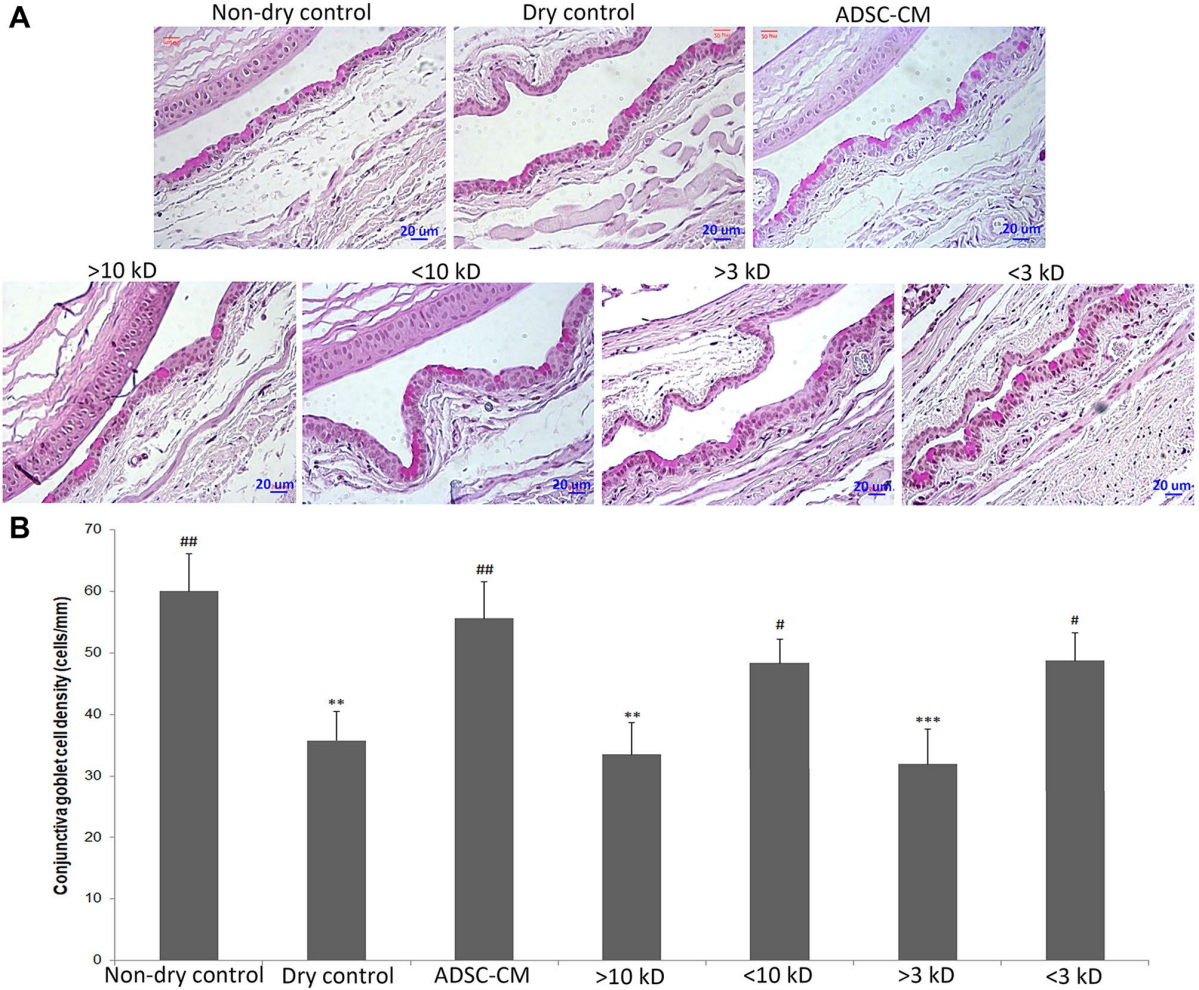
The conjunctival goblet cell density of the BALB/c mice in the controlled-environment chamber (CEC)-induced dry eye model. **(A)** The conjunctival goblet cells demonstrated by Periodic acid-Schiff stain were reduced in the controlled-environment chamber (CEC)-induced dry eye. Mice treated with ADSC-CM, fractions of < 10 kD, or < 3 kD, had higher goblet cell densities than those treated with > 10 kD and > 3 kD. The analytical data of the above were presented in **(B).** Data were in means ± SD. *p < 0.01, compared with Non-dry control. #p < 0.05, ##p < 0.01 compared with Dry control. (N = 3).

**Figure 6:**
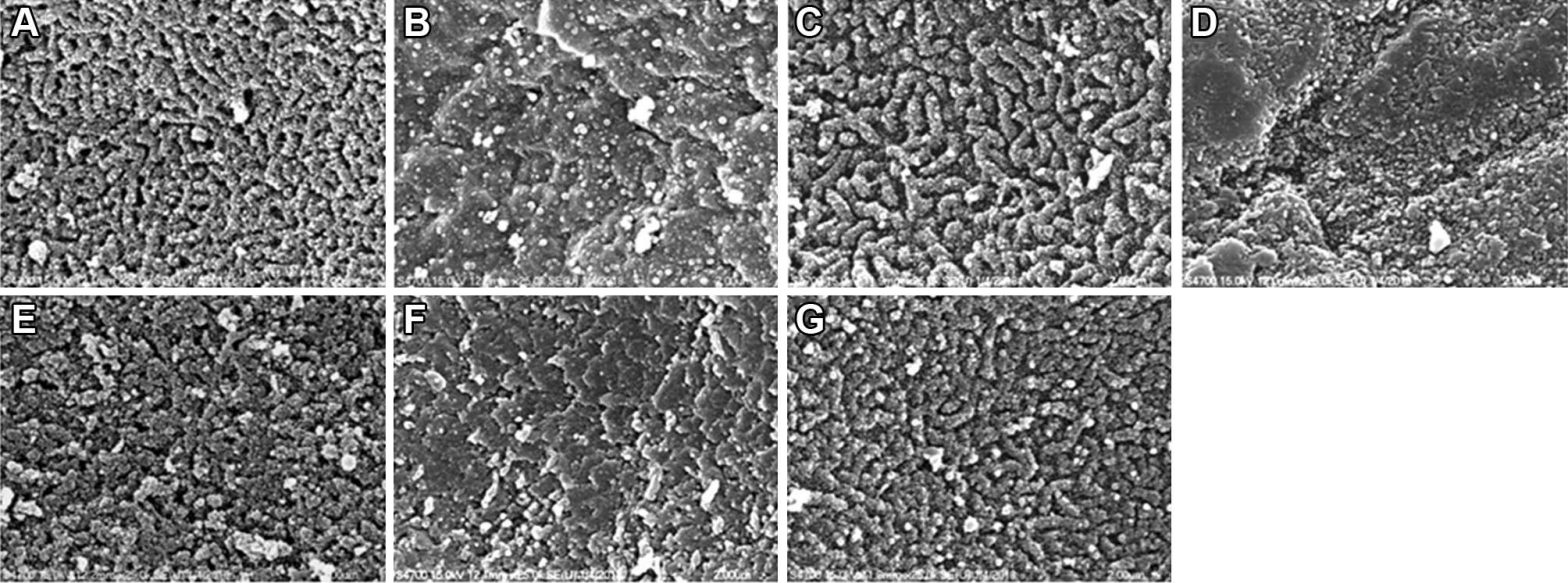
Scanning electron microscopy of cornea demonstrated that the microvilli of corneal epithelium were well-preserved in the non-dry control **(A)** but were mostly lost in the dry eye mice **(B)**. Topical application of ADSC-CM **(C)**, < 10 kD fraction of ADSC-CM **(E)**, or < 3 kD fraction of ADSC-CM **(G)** preserved the microvilli best from dry damage. In contrast, those treated with topical > 10 kD fraction of ADSC-CM **(D)** or > 3 kD **(F)** did not have good surfaces covered by microvilli. Magnification: ×25,000 (N = 3).

The second part of our study examined the effects of different size fractions of ADSC-CM in the dry eye via the in vitro human corneal epithelial cells (HCEC) desiccation stress study^54–58^ and the in vivo CEC mice dry eye model. ADSC-CM were fractionated into > 30 kD, < 30 kD, > 10 kD, < 10 kD, > 3 kD, and < 3 kD. The fractions of < 30 kD, < 10 kD, and < 3 kD provided better cell viabilities than those of > 30 kD, > 10 kD, and > 3 kD in the HCEC desiccation stress study (Fig. 3A). In the in vivo CEC mice dry eye experiment, mice treated with the fraction of < 10 kD and < 3 kD had higher tear secretion level (Fig. 3B), stronger expression of ZO-1 and MUC4 (Fig. 4), higher goblet cell density (Fig. 5), and better-preserved microvilli (Fig. 6).

**Figure 7:**
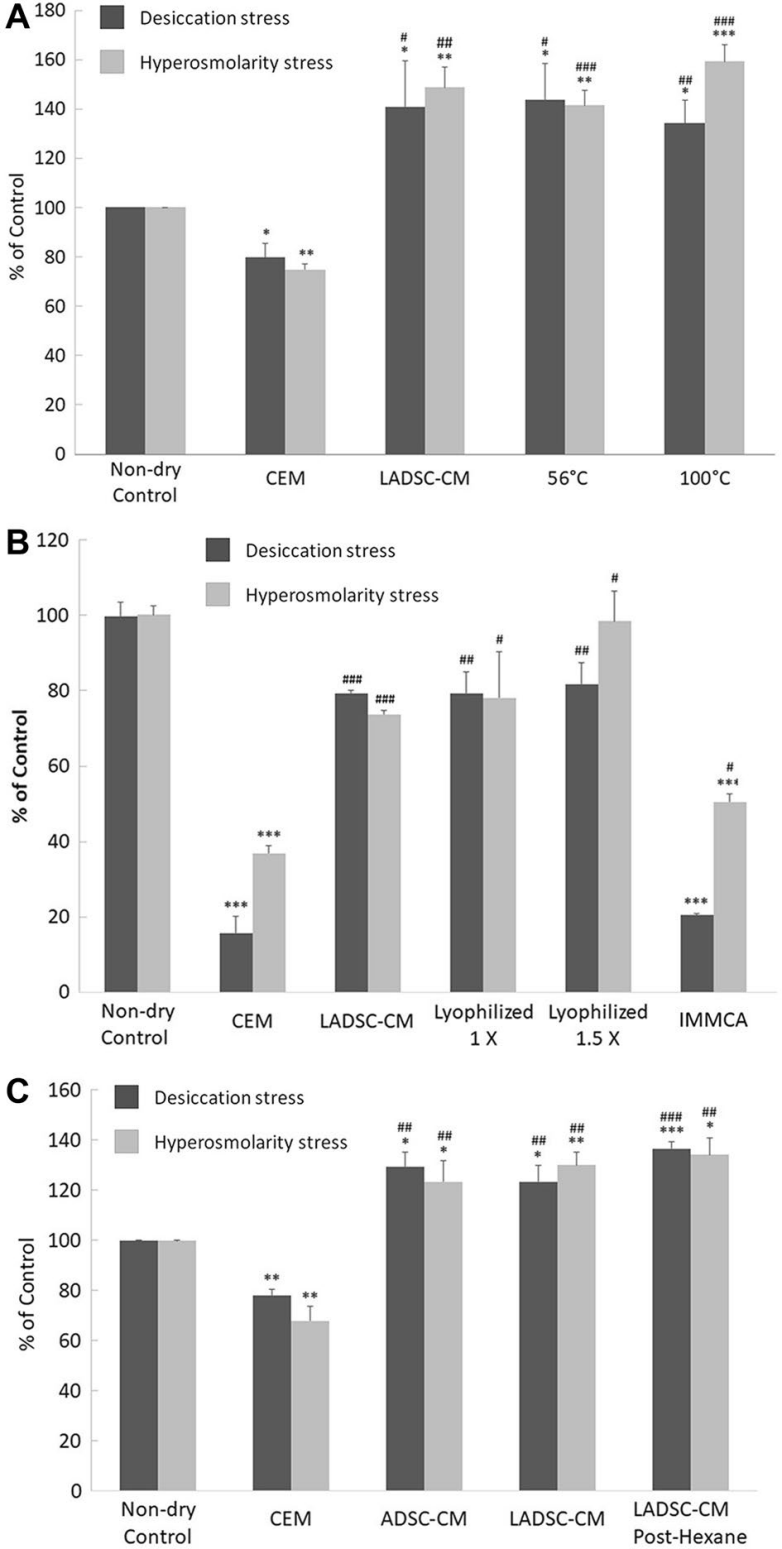
Protective effects of LADSC-CM on HCECs in desiccation stress or hyperosmolarity stress. **(A)** Heating to 56 °C for 30 min or 100 °C for 3 min did not reduce the protective effects of LADSC-CM. **(B)** The defensive capabilities after lyophilization, storage, and reconstitution were similar to those before the process. **(C)** An equal volume of hexane extraction three times did not change the protective effects of LADSC-CM (N = 5 in each experiment).

Further characterization of the active components in the LADSC-CM showed that heating to 56 °C for 30 min or 100 °C for 3 min did not reduce the defensive effects of LADSC-CM on HCECs against desiccating stress or hyperosmolarity stress. The protective capabilities did not lose after lyophilization, storage, and reconstitution. Neither did 1:1 hexane extraction for three times change the protective effects of LADSC-CM (Fig. 7).

## Discussion

Aging is one of the most critical factors in dry eye^59,60^, and antiaging approaches have been suggested for dry eye treatment^61,62^. Stem cells play essential roles against aging, but the application of stem cells might provoke the concerns of tumorigenesis or cellular rejection. In contrast, trophic factors in the conditioned medium secreted by stem cells also help tissue repair but minimize rejection problems as the conditioned medium is devoid of cells^63–67^. Besides, trophic factors in the conditioned medium might be freeze-dried or manufactured, packaged, and transported more easily. Therefore, stem cell-derived condition medium is promising as a pharmaceutical for regenerative medicine.

BAC has been used to induce dry eye in animals^51,52^, and the BAC-induced dry eye model was proved stable and widely used for research^31,53,68–75^. CEC-induced dry eye is another widely used model^48,76^. Disruption of tight junction, loss of conjunctival goblet cell, and impairment of membrane-associated mucin have been described in dry syndrome^77–79^. Previous studies described a similar finding in the BAC- or CEC-dry eye model^48,80–86^. In our study, the mice in the CEC treated with BAC concomitantly had significantly decreased expression of ZO-1 and occludin in the corneal epithelium. Topical application of ADSC-CM reversed or even increased the reduced expression. The corneal epithelium in the ADSC-CM group also showed the best presentation of K12, which meant preserving the characteristics of corneal epithelium^87^. The preservation of tight junction by ADSC-CM might help the corneal epithelium carry out housekeeping functions that border the external environment, including providing a barrier to fluid loss, toxin irritation, and pathogen entrance. Microvilli injury indicated by the surface covered by microvilli was suggested as the best determining indicator of progressive corneal exposure to dry eye conditions^88^. In our study, desiccation stress caused the loss of microvilli. The surface covered by microvilli was about 50 to 60% in the groups treated with Refresh Plus lubricant eye drops or IMMCA, while that in the ADSC-CM group was similar to that in the non-dry control group.

ADSC-CM protected not only the tight junction of corneal epithelial cells but also the conjunctival goblet cells and the membrane-associated mucin Muc16 expression. Conjunctival goblet cell density was an ocular biomarker of dry eye^89^. Muc16 was one of the major membrane-associated mucins expressed on the ocular surface epithelium^90^. Although Muc16 was expressed only in the conjunctival epithelia in mice^91^, in contrast to both the corneal and conjunctival epithelia in humans^92^, the loss of Muc16 in the conjunctiva affected the homeostasis of the corneal epithelium and stroma and upregulated the inflammatory signaling cascade^93^. In our study, the goblet cell density was decreased in the CEC mice, which was alleviated by Refresh Plus lubricant eye drops, IMMCA, or ADSC-CM. ADSC-CM protected the goblet cells density best to the level similar to that of non-dry mice. The Muc16 expression was continuous in the non-dry group and interrupted in the dry control group. ADSC-CM preserved the integrity of Muc16 expression from dry injury.

HCEC desiccation stress and hyperosmolarity stress are two widely used in vitro models to simulate dry eye conditions^55,94–100^. In the HCEC desiccation stress study, ADSC-CM showed better protective or regenerative effect than the commercial corneal epithelial basal medium CEM, with the fractions of < 30 kD, < 10 kD, and < 3 kD providing better cell viabilities than those of > 30 kD, > 10 kD, and > 3 kD. From above, we believe something in LADSC-CM (< 3 kD) beneficial for dry eye and are exploring the possible underlying mechanism.

Topical application of human ADSC-derived exosomes (size about 100 nM) has recently been reported to alleviate ocular surface damage in a mouse model of dry eye disease^47^. In contrast, our study showed better protective effects of the low-molecule-weight (< 3 kD, less than 0.6 nM; < 10 kD, less than 1.5 nM ) fractions of ADSC-CM (LADSC-CM). Mice treated with LADSC-CM had more tear secretion, well-preserved tight junction and MUC16 expression of the cornea, higher conjunctival goblet cell density, and less damaged microvilli of corneal epithelium. LADSC-CM contained only molecules smaller than the known smallest virus that is in the size of about 15 nM, making its clinical application free from infection risk. The protective capabilities remain similar after heating to 56 °C for 30 min or 100 °C for 3 min, or lyophilization and reconstitution, which meant the active ingredients were relatively stable and might be easier for transportation. The active components in LADSC-CM were resistant to hexane extractions and were probably polar in characters.

In conclusion, our study demonstrated the beneficial effects of LADSC-CM in both the in vitro HCEC desiccation stress study and in vivo mice dry-induced ocular surface injury. The active ingredients might be stable and polar molecules. Further investigation of the exact active ingredient(s) and the underlying mechanism is needed.

## Methods

### Isolation of ADSCs and preparation of ADSC-CM

This study was approved by the Buddhist Tzu Chi General Hospital Internal Review Board (IRB102-130). All methods were performed in accordance with the relevant guidelines and regulations. Written informed consent was obtained from all participants. Human adipose tissue was harvested during cosmetic liposuction from abdominal subcutaneous fat of three women (age: 23, 28, and 30). Stromal-vascular fraction (SVF) cells were isolated using a modified method described by Griesche and colleagues^101^. Collagenase type I (final concentration: 0.4 mg/mL; Sigma) was added for enzymatic digestion in a hybridization oven (37 °C, 30° angle, 15 rpm, 45 min). Digested adipose tissue was centrifuged at 400 × g for 10 min to generate the SVF pellets for subsequent ADSCs culture. The stemness of the ADSCs was confirmed by their osteogenesis, chondrogenesis, and adipogenesis after induction. ADSCs at passages 2 to 5 were cultured in non-Phenol Red Iscove’s modified Dulbecco’s medium (Gibco™) supplemented with glutamine (200 mM; Gibco™), 10% FBS (HyClone™) and mesenchymal stem cells culture adjuvant (FGF2, 10 ng/ml, R&D Systems; N-acetyl-L-cysteine, 2 mM, Sigma; L-ascorbic acid-2-phosphate, 0.2 mM, Sigma) (The medium was abbreviated as IMMCA). Conditioned mediums were collected after 72 h of culture and mixed, centrifuged at 300 × *g* for 5 min, filtered through 0.22 μm syringe filter, aliquoted, and frozen for experimental use.

### Preparation of different size fractions of ADSC-CM

Collected ADSC-CM was added to 30 kDa, 10 kDa, or 3 kDa Amicon ultra-15 centrifugal filter tube (Millipore, Billerica, USA) or Spectrum^®^ hollow fiber filter (Repligen, Boston, USA and) and centrifuged at 4000 *g*. The supernatants (> 30 kDa, > 10 kDa, > 3 kDa, respectively) were diluted with IMDM to their initial concentration. The filtered fluid (< 30 kDa, < 10 kDa, < 3 kDa) were also collected for experiments.

### Characterization of active components in the LADSC-CM

For the heating test, LADSC-CM was incubated at either 56 °C for 30 min or 100 °C for 3 min and was tested for activity. For the lyophilization test, aliquots of the dialyzed LADSC-CM samples (1 ml) were prepared in 5 ml lyophilized vials followed by lyophilization in a programmable freeze dryer. The lyophilized products were stored at 4 °C for one week and were then reconstituted with water for injection for activity test. For the lipophilicity test, equal volumes of LADSC-CM and hexane (3 mL for each) were mixed and vortexed for 20 min and centrifuged at 2000 rpm for 5 min, and the hexane fraction was discarded. The extraction was repeated three times, the lower layer (aqueous phase) was collected for test.

### Human corneal epithelial cells (HCECs) culture

Normal primary HCECs from American Type Culture Collection (ATCC^®^, Manassas, VA, USA) were maintained according to the instructions. The HCECs were grown in a corneal epithelial cell basal medium supplemented with corneal epithelial cell growth kit components (CEM, ATCC^®^). The cells were cultured at 37 °C in a moist atmosphere with 5% carbon dioxide. The culture medium was changed every 2 or 3 days. In this study, only sub-confluent HCECs at passage three were used.

### Desiccating stress

A modified in vitro desiccation stress on HCECs was used in our study^55,57,96^. Briefly, 2 × 10^4^ HCECs were seeded in 96-well dishes and cultivated for 24 h to attach to the dishes (about 80% confluence). The medium was aspirated, and the dishes were left dry for 10 min at 37 °C. After desiccation, the testing culture mediums were replenished to the respective culture dishes. The HCECs that did not undergo the desiccation stress were deemed as control. After incubation for four hours, the cells were counted using the Cell Counting Kit-8 (CCK-8 assay).

### Hyperosmolarity stress

1.5 × 10^4^ HCECs were seeded in 96 well-dishes and cultivated overnight to attach to the dishes (approximately 60% confluence) and were then treated for 24 h with fresh medium (311 mOsm/kg, normal control) or the medium containing another 90 mM NaCl (490 mOsm/kg, hypertonic groups). After Hypertonic treatment, the cells were cultured in respective testing culture mediums. The cells were estimated using a CCK-8 assay after 4 h of incubation.

### CCK-8 assay

Cell viability was measured using Cell Counting Kit-8 (CCK-8; Enzo Life Sciences, Farmingdale, NY, USA). 10 μl CCK-8 reagent was added to cells grown on a 96-well culture plate containing 100 μl culture media. After incubation at 37 °C for 3 h, the cells were estimated via absorbance at 450 nm using a microplate reader (MicroQuant, BioTek Instruments, Inc., Winooski, VT, USA).

### Induced murine dry eye model

The study is reported in accordance with ARRIVE guidelines (https://arriveguidelines.org). All experimental procedures were approved by the Laboratory Animal Care and Use Committee at Tzu Chi University. All methods were performed in accordance with the relevant guidelines and regulations. Dry eye-related ocular surface signs of BALB/c mice were induced in a controlled-environment chamber (CEC)^48–50^ combined with topical BAC^51–53^. Briefly, 12-week-old female BALB/c mice were housed in CEC with a relative humidity of 10 ± 3%, temperatures of 21–25℃, and airflows of 10–15 L/min. Each experimental and control group consisted of 5 mice. Mice of dry control and experimental groups were housed in the CECs and received topical 0.2% BAC daily. Mice of the non-dry control group were in a chamber of humidity of 75 ± 3%. The experimental groups received the respective testing eye drops twice a day for 28 days. In the first experiment, the testing eye drops were Refresh Plus lubricant eye drops, IMMCA, and ADSC-CM. In the second part experiment, the testing eye drops were the original ADSC-CM, ADSC-CM with a molecular weight > 10 KD, < 10 KD, > 3 KD, and < 3 KD, respectively. Tear secretion assay was performed weekly. The mice were sacrificed with overdoses of pentobarbital at the end of the experiments (28^th^ day), and the eyeballs were harvested for histological and immunohistochemical study and scanning electric microscopic examination.

### Tear secretion assay

Tear secretion was estimated by the length of the tear-absorbed, color-changed region on Zone-Quick phenol red thread (Showa Yakuhin Kako Co., LTD., Japan). Briefly, the excess tears were removed for a standard time of 4 s, and the Zone-Quick phenol red threads were then held with jeweler forceps and placed in the lateral fornix for 30 s. The left eyes were measured first and then the right eyes. The average of both eyes was used for analysis.

### Histological analysis

The eyes and ocular adnexa were fixed in 10% formaldehyde and embedded in paraffin. Central vertical plane sections of 3 μm thickness were stained with hematoxylin–eosin or Periodic acid-Schiff. The densities of conjunctival goblet cells were calculated using the *ImageJ* assay.

### Immunohistochemistry

The eyes were fixed in 10% formaldehyde. After paraffin embedding, 3-μm-thick sections were dewaxed in xylene, rehydrated in a series of ethanol solutions, and washed twice in distilled water. Antigen retrieval was performed with Dako Target Retrieval Solution pH 9 (Dako, Glostrup, Denmark) for 15 min at 90–95 ˚C. Sections were blocked with 1% BSA in PBS with 0.3% Triton X-100 for at least 1 h at room temperature. The slides were incubated with the rabbit anti-ZO-1(Mid) (1:100; Invitrogen, Camarillo, CA, USA), mouse anti-occludin (1:50; Thermo Scientific, Rockford, IL, USA), or goat anti-cytokeratin 12 (1:50; Santa Cruz, Santa Cruz, CA, USA) overnight at 4 °C, followed by Alexa Fluor 488 donkey anti-rabbit IgG (H + L) (1:800; Jackson ImmunoResearch, West Grove, PA, USA), Dylight 550-conjugated goat anti-mouse IgG (H + L) or Dylight 550-conjugated donkey anti-goat IgG (H + L) (1:500; Bethyl Laboratories, Montgomery, TX, USA) for 1 h at room temperature. The nucleus was counterstained with 4’,6’-diamidino-2-phenylindole (DAPI; Molecular Probes, Eugene, OR, USA). The slides were mounted and examined with a Zeiss LSM 510 META confocal microscope. In negative controls, the primary antibody was substituted with the blocking buffer.

MUC16 staining was performed on 8-μm-thick sections using Histofine Mouse Stain Kit (Nichirei, Tokyo, Japan). The sections were incubated with mouse anti-MUC16 (1:50; Santa Cruz, Santa Cruz, CA, USA) overnight at 4 °C, and finally with Histofine Simple Stain Max PO for 10 min. The horseradish peroxidase reaction was developed with 3,3′-diaminobenzidine tetrahydrochloride w/Co (D-0426, Sigma, Saint Louise, Missouri, USA). Negative control studies were also performed without using the primary antibodies. After dehydration in graded ethanol and xylene, sections were mounted in Histokit (Hecht Assistent, Sondheim, Germany) and analyzed.

### Scanning electron microscopy analysis

Fresh corneas were first fixed in 2% paraformaldehyde for 24 h, and then in 2.5% glutaraldehyde solution in 0.2 M cacodylate buffer and 1% tannic acid at pH 7.0–7.3 for another 24 h, followed by postfixation with 1% osmium tetroxide solution in 0.2 M cacodylate buffer solution for 1 h. Samples were then dehydrated by a critical point dryer (Hitachi Ltd., Japan) and coated with platinum in an ion sputter coater (Hitachi Ltd., Japan). Finally, the samples were observed and photographed with the scanning electron microscope (Hitachi Ltd., Japan).

### Statistical analysis

Data were expressed as means ± SD. Only one sample from each mouse was used for the analysis of each examination result. In the tear secretion assay, the average of estimates from both eyes was used. For goblet cell density, only the left eye of each mouse was sectioned for Periodic acid-Schiff stain and calculation. For CCK viability assay, each sample in the same group was from different rounds of experiments. One-way ANOVA and two-sample *t*-test were used to compare CCK assay, tear secretion assay, and conjunctival goblet cell density. A p < 0.05 was considered statistically significant.
